# Angiotensin II Modulates Calcium/Phosphate Excretion in Experimental Model of Hypertension: Focus on Bone

**DOI:** 10.3390/biomedicines10112928

**Published:** 2022-11-14

**Authors:** Giovanna Castoldi, Raffaella Carletti, Silvia Ippolito, Isabella Villa, Biagio Palmisano, Simona Bolamperti, Alessandro Rubinacci, Gianpaolo Zerbini, Michela Meani, Giovanni Zatti, Cira R. T. di Gioia

**Affiliations:** 1 Dipartimento di Medicina e Chirurgia, Università degli Studi di Milano-Bicocca, 20900 Monza, Italy; 2 Dipartimento di Medicina Traslazionale e di Precisione, Sapienza Università di Roma, 00185 Rome, Italy; 3 Laboratorio Analisi Chimico Cliniche, Ospedale San Gerardo, ASST Monza, 20900 Monza, Italy; 4 Laboratorio di Endocrinologia e Metabolismo Osseo, Istituto di Endocrinologia e Scienze Metaboliche, IRCCS Ospedale San Raffaele, 20132 Milano, Italy; 5 Dipartimento di Medicina Molecolare, Sapienza Università di Roma, 00161 Roma, Italy; 6 Osteoporosis and Bone and Mineral Metabolism Unit, IRCCS Ospedale San Raffaele, 20132 Milano, Italy; 7 Unita’ Complicanze del Diabete, IRCCS Ospedale San Raffaele, 20132 Milano, Italy; 8 Clinica Ortopedica, Ospedale San Gerardo, ASST Monza, 20900 Monza, Italy; 9 Dipartimento di Scienze Radiologiche, Oncologiche e Anatomopatologiche, Istituto di Anatomia Patologica, Sapienza Università di Roma, 00161 Rome, Italy

**Keywords:** angiotensin II, experimental hypertension, bone, rats

## Abstract

A link between hypertension and long-term bone health has been suggested. The aim of this study was to investigate the effects of chronic angiotensin II administration on urinary calcium/phosphate excretion, bone mineral density, bone remodeling and osteoblast population in a well-established experimental model of hypertension, in the absence of possible confounding factors that could affect bone metabolism. Male Sprague–Dawley rats, divided in the following groups: (a) Angiotensin II (Ang II, 200 ng/kg/min, osmotic minipumps, sub cutis, *n* = 8); (b) Ang II+losartan (Los, 50 mg/kg/day, *per os*, *n* = 6); (c) control group (physiological saline, sub cutis, *n* = 9); and (d) control+losartan (*n* = 6) were treated for four weeks. During the experimental period, 24-hour diuresis, urinary calcium, phosphate and sodium excretion were measured prior to the treatment, at two weeks of treatment, and at the end of the treatment. Systolic blood pressure was measured by plethysmography technique (tail cuff method). At the end of the experimental protocol, the rats were euthanized and peripheral quantitative computed tomography at the proximal metaphysis and at the diaphysis of the tibiae and quantitative bone histomorphometry on distal femora were performed. Angiotensin II-dependent hypertension is associated with increased calcium and phosphate excretion. AT1 receptor blockade prevented the increase of blood pressure and phosphate excretion but did not affect the increase of calcium excretion. These changes took place without significantly affecting bone density, bone histology or osteoblast population. In conclusion, in our experimental conditions, angiotensin II-dependent hypertension gave rise to an increased urinary excretion of calcium and phosphate without affecting bone density.

## 1. Introduction

Epidemiologic data suggest a possible link between arterial hypertension, characterized by cardiovascular and renal complications, and a degenerative disease of the bone, known as osteoporosis [1,2,3,4]. Both arterial hypertension and osteoporosis show increased frequency in aging, especially in post-menopausal women, and share the same risk factors, such as low physical activity or smoking. These two diseases have similar pathophysiological mechanisms, such as increased oxidative stress, inflammation, and dysfunctions of calcium homeostasis [5,6,7,8,9].

Systemic renin-angiotensin system (RAS) has a fundamental role in the regulation of blood pressure and electrolyte balance [10,11], and Angiotensin II (Ang II) has been historically considered the main effector of RAS. Besides the ‘classical’ systemic RAS, it has been demonstrated that the components of RAS are expressed in multiple tissues [12]. At the same time, Ang II, in addition to its well-known hemodynamic action, has many cellular effects in different tissues promoting inflammation [13,14,15], fibrosis [13,16,17], and hypertrophy [17,18,19], mainly mediated by AT1 receptors. 

Bone tissue expresses the components of RAS which are involved in bone remodeling [20,21,22]. In fact, experimental studies have demonstrated that Ang II increases the activity of osteoclasts, enhancing bone loss in rats affected by osteoporosis caused by estrogen deficiency [23]. A role for bone RAS has been described in age-related osteoporosis in mice [24] and in the reduction of bone formation and in the increase of resorption in obstructive nephropathy [25]. 

AT1 receptor knockout mice showed an increase in bone mass [26], possibly suggesting a protective role of RAS inhibition in bone. However, the inhibitory effects of captopril on bone RAS components did not improve bone damage caused by type II diabetes in db/db mice [27]. In type 1 diabetes mice (streptozotocin induced) the increased activity of bone RAS was shown to be involved in osteoporosis processes, which was not improved by ACE inhibitors [28]. Finally, in ovariectomized rats, the stimulation of the protective arm of RAS (ACE-2/Ang 1-7/Mas) was shown to mediate the osteo-protective effects of RAS inhibition [21].

These data strongly suggest that the role of RAS in bone remodeling might differ depending on the underlying pathological conditions, such as estrogen deficiency, hypertension, diabetes and osteoporosis.

Nevertheless, the direct effects of angiotensin II dependent hypertension on bone tissue are not completely understood. 

In the present study we investigated the effects of chronic angiotensin II administration on urinary calcium/phosphate excretion, bone mineral density, bone remodeling and on osteoblast population—the cells responsible for the synthesis of bone matrix—in a well-established experimental model of hypertension, in the absence of possible confounding factors that could affect bone metabolism.

In this study we demonstrate that angiotensin II-dependent hypertension is associated with a modulation of urinary calcium/phosphate excretion over time leading to an increase of calcium and phosphate excretion. AT1 receptor blockade prevented the increase of blood pressure and phosphate excretion but did not affect the increase of calcium excretion. These changes took place without significantly affecting bone density, bone histology, and osteoblast population.

## 2. Materials and Methods

### 2.1. Animal Study Design of Ang II-Dependent Hypertension

Experiments were performed in male Sprague–Dawley rats (body weight 150–175 g) in accordance with the Guide for the Care and Use of Laboratory Animals published by the US National Institutes of Health (NIH Publication No. 85–23, revised 1996). Animal husbandry was in conformity with the institutional guidelines in compliance with national laws and policies (D.L.n. 116, Gazzetta Ufficiale della Repubblica Italiana, suppl.40, 18 February 1992). Rats were individually housed in cages (or metabolic cages as necessary) in a temperature-controlled room with a 12:12 light:dark cycle, with free access to a standard rat chow and tap water. One week before the beginning of the protocol, rats were accustomed to metabolic cages and experimental procedures. Body weight (BW, g) was measured once a week. Systolic blood pressure was measured by plethysmography technique (tail cuff method, average of six recordings using a BP Recorder (Ugo Basile Instruments, Varese, Italy)) at the beginning and at the end of the experimental protocols [29,30]. Ang II was administrated through osmotic minipumps (Alzet 2004, Palo Alto, CA, USA), subcutaneously implanted under sodium pentobarbital anesthesia (40 mg/kg/i.p.), in order to administer Ang II (200 ng/Kg/min for 4 weeks, *n* = 8, Sigma) or physiological saline in the control group (*n* = 9). Losartan (Los, 50 mg/kg/day), dissolved in drinking water, was administered in control + Los (*n* = 6) and Ang II + Los treated rats (*n* = 6). Glomerular filtration rate (mL/min) was evaluated as creatinine clearance using 24 h diuresis (ml/24 h).

Non-fasting plasma glucose (mg/dL), creatinine (mg/dL), sodium (mEq/L), potassium (mEq/L), calcium (mg/dL), phosphate (mg/dL), alkaline phosphatase (U/L), uric acid (mg/dL), total cholesterol (mg/dL), triglycerides (mg/dL) and 24 h urinary calcium (mg/24 h), phosphate (mg/24 h), and sodium (mEq/24 h) were measured by colorimetric technique on Cobas Roche (Mannheim, Germany) [15]. 

At the end of the experimental periods, rats were euthanized by an overdose of anesthesia. Hearts and kidneys were excised and weighted. The femurs were excised and fixed with 10% formalin, embedded in paraffin and used for light microscopic examination and morphometric analysis. Contralateral femur with tibia was also dissected, fixed with 10% formalin solution, neutral buffered, and used for peripheral quantitative computed tomography.

### 2.2. Peripheral Quantitative Computed Tomography (pQCT) Analysis

The measurements were obtained by using a Stratec Research SA+ pQCT scanner (Stratec Medizintechnik GmbH, Pforzheim, Germany) with a voxel size of 70 mm and a scan speed of 3 mm/s. The excised tibiae were hold in place with manufacturer-made plastic holders in order to position the long axes of the specimen parallel to the image planes. The correct longitudinal positioning was resolved by means of a “scout scan”. The scans were performed at the proximal metaphysis and at the diaphysis of the tibiae. The scans were analyzed with pQCT software 6.00B using contour mode 2 and peel mode 2. The threshold for the calculation of trabecular and total bone parameters was 500 mg/cm^3^ and for cortical bone parameters 710 mg/cm^3^.

### 2.3. Bone Histology and Histomorphometry

For each rat the formalin fixed femur was decalcified in 10% EDTA for 30–45 days at 4 °C with gentle shaking and processed for paraffin embedding. Three-micron-thick paraffin embedded sections were used for standard histology after staining with Hematoxylin-Eosin (H&E) or with Sirius red to visualize collagen fibers with morphometric analysis.

Quantitative bone histomorphometry was conducted on distal femora. Experiments were performed in a blinded fashion. The region of interest (ROI) was identified in the secondary spongiosa of distal femora, starting 500 μm below the growth plate and for a length of 5 mm. Sections were stained with H&E and Sirius red and used to measure trabecular bone volume per tissue volume (BV/TV), osteoblast number per bone surface (N.Ob/BS) and osteoblast surface per bone surface (Ob.S/BS). Ten pictures at 20X were acquired with an optical microscope (Leica DMRB, Leica Biosystems, Muttenz, Switzerland) through a digital camera (Leica LAS EZ, Leica Biosystems, Switzerland) and all histomorphometric analyses were performed using Image J [31].

### 2.4. Statistical Analysis 

Data are reported as means ± standard error of the mean (SEM). Differences among the groups of rats (control, control+losartan, Ang II, Ang II+losartan) for systolic blood pressure, body weight, glomerular filtration rate, heart/body weight, kidney/body weight, blood glucose, alkaline phosphatase, sodium, potassium, calcium, phosphate, uric acid, cholesterol and triglycerides, 24 h diuresis, 24 h urinary calcium, phosphate and sodium excretion, pQCT parameters, trabecular bone volume per tissue volume (BV/TV), osteoblast number per bone surface (N.Ob/BS) and osteoblast surface per bone surface (Ob.S/BS) were assessed using ANOVA followed by Fisher’s protected least-significant test for post hoc comparisons. Differences between means were considered significant at *p* < 0.05.

## 3. Results

### 3.1. Effects of Ang II and Losartan Administration on Systolic Blood Pressure, Body Weight, Glomerular Filtration Rate, Heart/Body Weight Ratio, Kidney/Body Weight Ratio and Serological Parameters

Ang II administration caused a marked increase in blood pressure, which was prevented by losartan treatment in Ang II treated rats (Table 1). Losartan administration did not significantly modify blood pressure in control rats (Table 1). 

As compared to control rats, Ang II administration caused a significant reduction in BW, which was blunted by losartan treatment (Table 1). Ang II administration caused a slight decrease in glomerular filtration rate as compared to control rats. Losartan treatment prevented the decrease of GFR in Ang II treated rats (Table 1).

Ang II administration caused a significant increase in the heart/BW and kidney/BW ratios as compared to control rats, in line with the possible development of myocardial and kidney hypertrophy/fibrosis [15,16,17]. Losartan administration prevented the increase of heart and kidney/BW ratio in Ang II treated rats (Table 1).

As shown in Table 1, Ang II administration caused a slight decrease in plasma sodium, potassium and phosphate values as compared to control rats, which was prevented by losartan treatment in Ang II treated rats. No significant changes in plasma alkaline phosphatase, calcium, uric acid and in glyco-metabolic parameters (non-fasting glucose, cholesterol, triglycerides) were observed among the different groups (Table 1).

### 3.2. Effects of Ang II and Losartan Administration on 24 h Diuresis, Urinary Sodium, Calcium, and Phosphate Excretion

**Figure 1:**
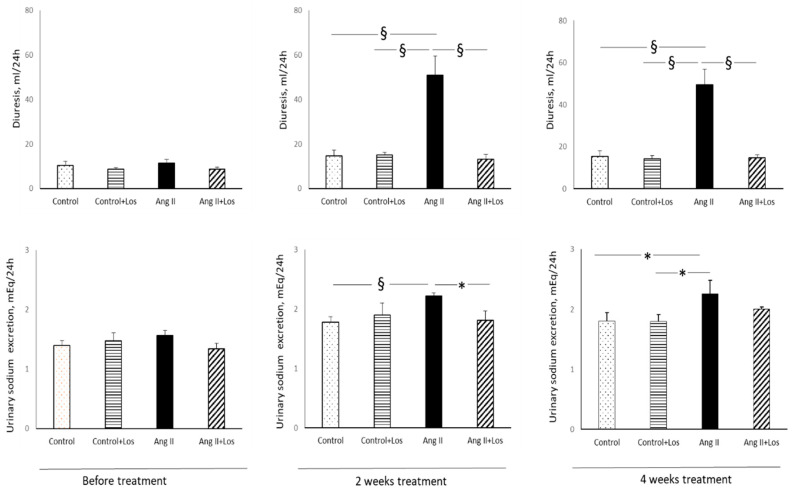
Effects of Ang II and losartan administration on 24 h diuresis and urinary sodium excretion before and after two and four weeks of different treatments. Data are means ± SEM. * = *p* < 0.05; § = *p* < 0.01.

**Figure 2:**
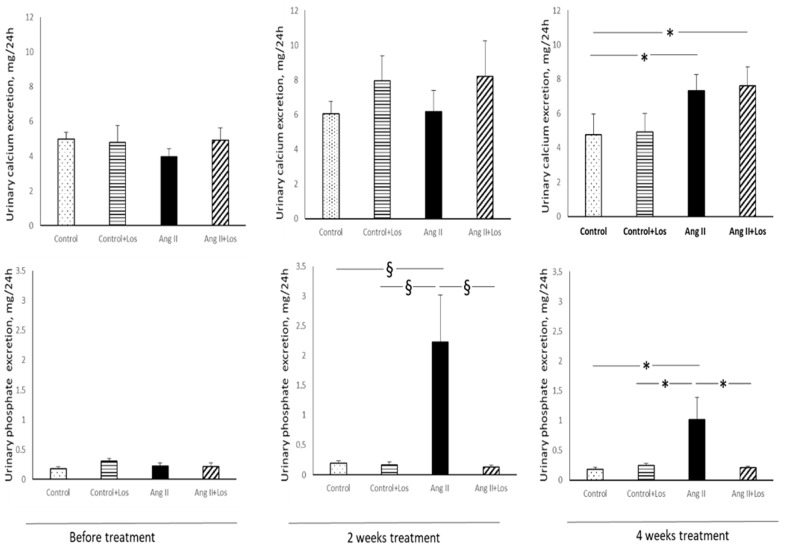
Effects of Ang II and losartan administration on 24 h urinary calcium and phosphate excretion before and after two and four weeks of different treatments. Data are means ± SEM. * = *p* < 0.05; § = *p* < 0.01.

Before starting the treatments, diuresis and urinary sodium, calcium, and phosphate excretion were similar in all the groups (Figure 1 and Figure 2).

Ang II administration increased diuresis just after two weeks of administration and until the end of the experimental period as compared to other groups (Figure 1). Losartan treatment blocked the increase in diuresis in Ang II treated rats during the entire experimental period (Figure 1).

After two weeks of Ang II administration and until the end of the experimental protocol Ang II administration caused a significant increase in urinary sodium excretion as compared to control rats (Figure 1). Losartan treatment blocked the increase in urinary sodium excretion in Ang II-treated rats (Figure 1).

After two weeks of Ang II administration no significant changes in urinary calcium excretion were observed in Ang II treated rats, as compared to other groups (Figure 2).

At the end of the experimental period, treatment with Ang II, alone or in combination with losartan, induced an increase of urinary calcium excretion as compared to control rats (Figure 2). Ang II administration increased phosphaturia just after two weeks of administration and until the end of the experimental period as compared to control rats (Figure 2). Otherwise, losartan treatment blocked the increase in urinary phosphate excretion in Ang II treated rats just after two weeks of administration until the end of experimental protocol (Figure 2).

### 3.3. pQCT Analysis of Bone Parameters after Four Weeks of Treatment with Ang II and Losartan

**Figure 3:**
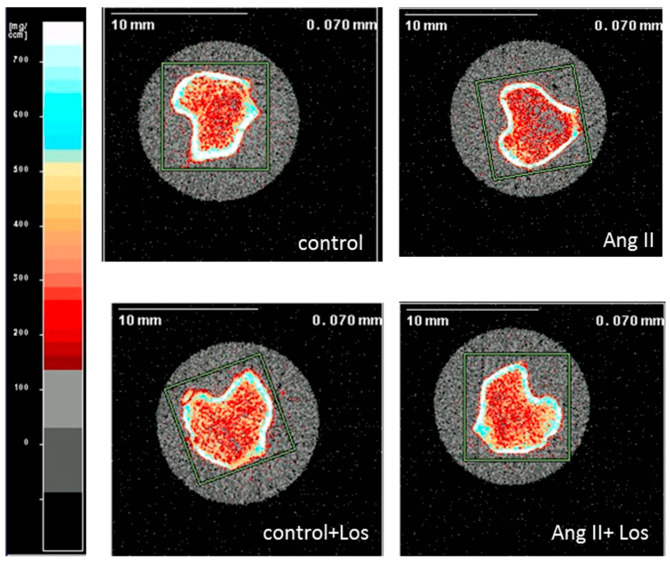
Representative image of the metaphyseal site of the rat tibiae analyzed by pQCT. The bar on the left is the colour scale bar of the density (mg/cm^3^): white > 700 mg/cm^3^; grey ≤ 100 mg/cm^3^.

After four weeks of treatment, Ang II did not modify any bone parameters compared to control measured at the proximal metaphysis and at the diaphysis midshaft of the tibiae. Losartan induced an increase in Tb.BMD in rats chronically treated with Ang II and in control rats, although not reaching statistical significance (Table 2, Figure 3).

### 3.4. Histomorphometric Analysis of Bone Parameters after Four Weeks of Treatment with Ang II and Losartan

**Figure 4:**
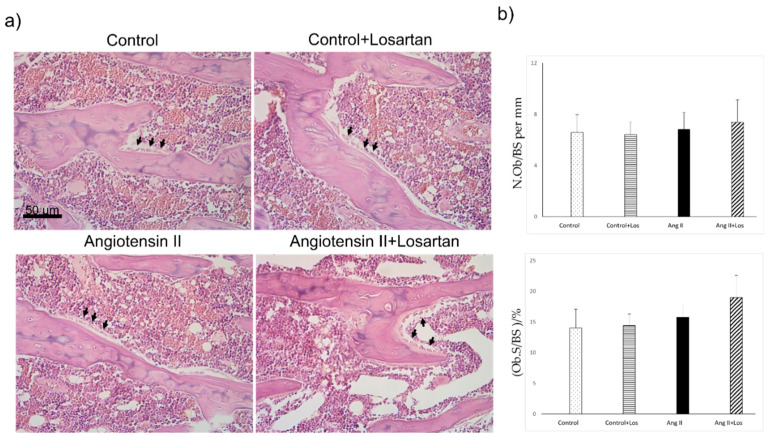
Effects of Ang II and Losartan administration on bone tissue. (**a**) Representative images from H&E-stained sections showing osteoblasts (arrow) and (**b**) quantification of osteoblast parameters in the different groups of rats. Data are means ± SEM. N.Ob: Osteoblast number; BS: bone surface; Ob.S: osteoblast surface; BS: bone surface. Ang II: Angiotensin II; Los: losartan.

Osteoblast parameters, quantified by morphology and histomorphometry and normalized for trabecular bone surfaces (N.Ob/BS and Ob.S/BS) did not differ between Ang II treated rats and control animals. Losartan treatment did not modify osteoblast number/bone surface and osteoblast surface/bone surface in control and Ang II treated rats (Figure 4).

**Figure 5:**
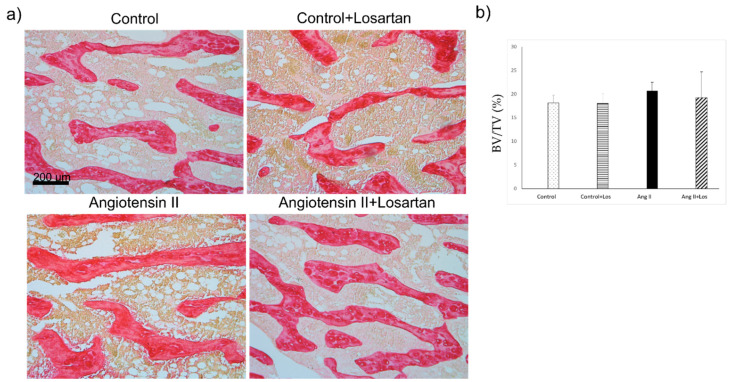
Effects of Ang II and losartan administration on bone tissue. (**a**) Representative histological pictures from Sirius red-stained bone tissue sections (trabecular bone stained in red) and (**b**) quantification of femoral trabecular bone in the different groups of rats. BV: Trabecular bone volume; TV: tissue volume. Data are means ± SEM. Ang II: Angiotensin II; Los: losartan.

Trabecular bone volume of femora in Ang II treated rats did not show significant difference as compared with control groups and Ang II+losartan treated rats (Figure 5).

## 4. Discussion

The results of this study demonstrate that chronic Ang II administration caused an early increase in urinary excretion of calcium and phosphate that lasted a long time. The increase was paralleled by a decrease in plasma phosphate levels, while plasma calcium levels did not change. 

The blockade of AT1 receptors by losartan treatment prevented the increase of phosphaturia, but not the increase in urinary calcium excretion. 

A link between hypertension and bone metabolism has been described in the literature, in particular a relationship between high blood pressure and hypercalciuria, which may promote bone mineral loss [32,33,34].

In our case the Ang II-induced increase of calcium and phosphate excretion is not associated with changes in bone mass. 

The observed enhancement of renal phosphate loss in Ang II treated animals might be primarily related to a direct effect of Ang II on the type II sodium-phosphate co-transporter (NaPi-IIa) that is the critical player in renal Pi regulation. It has indeed been shown that phosphate excretion increased seven fold in rats chronically treated with Ang II with an associated enhancement, likely induced by post-transcriptional mechanisms, of NaPi-IIa protein level with Western blot analysis of the brush border membrane vesicles exposed to Ang II [35]. This effect is potentially mediated by the Ang II type 1 receptor, and, in line with our experimental model, losartan prevented the increase of phosphate excretion in Ang II treated rats.

Beyond the direct effect of Ang II on NaPi-IIa, it should be considered that the endocrine regulators of the renal tubular maximum phosphate reabsorption (TmP) might be affected by RAS activation and subsequently constitute an endocrine milieu favoring kidney phosphate loss. In fact, the increased (sodium-driven) calcium excretion induced by Ang II administration could give rise to a negative calcium balance with secondary activation of parathyroid hormone (PTH). This phenomenon would result, just as in our case, an increased urinary excretion of phosphate and a decrease in plasma phosphate. PTH is in fact a known regulator of TmP and could directly contribute to bone and heart damage. PTH secretion could also be directly enhanced by aldosterone [36] that can in turn be stimulated by Ang II. This assumption is supported by the evidence described in the literature that RAS inhibitors lower PTH [37,38]. 

Taking into account the extent of the effects on phosphate metabolism in relation to the minor effects on calcium, it is also possible, as an additional hypothesis, that the increased urinary excretion of phosphate and reduced plasma phosphate levels are related to the activation of Fibroblast Growth Factor23 (FGF23) secretion by Ang II and/or aldosterone [39]. It has indeed been shown that Ang II and aldosterone stimulate FGF23 transcription and secretion in cultured osteoblast-like cells [40] and cardiac myocytes [41]. FGF23 is a 251-amino-acid long, primarily bone derived, hormone that is critical to the maintenance of phosphate homeostasis [42]. In the proximal kidney tubule, FGF23 binds to the FGF receptor (FGFR) and its co-receptor klotho and downregulates the membrane availability of the phosphate type II sodium-phosphate co-transporter, NaPi-IIa. This effect, acting in a combined manner with PTH signaling [43], results in increased urinary excretion of phosphate and reduced plasma phosphate levels. The interactions among PTH, FGF23 and RAS signaling on NaPi-IIa might therefore constitute the endocrine milieu which drives the phosphate loss in a synergistic manner, although the specific contribution of each regulatory signal remains unclear. If this was the case, the activation of FGF23 could at the same time participate in cardiac damage. In fact, Ang II inhibition has been shown to reduce FGF23-induced changes in Ca2+ homeostasis and hypertrophy in cardiac cells [44]

Interestingly, in our experimental conditions, an increase in urinary calcium excretion, but not an increase in urinary phosphate excretion, was present in Ang II+losartan treated group, in the absence of both hypertension and increased urinary sodium excretion. Whether this increase is a transitory effect or may last a long time, mediated by other mechanisms not related to the blockade of AT1 receptors or by the increase in blood pressure caused by Ang II, remains to be clarified.

However, in our experimental model of Ang II dependent hypertension we did not find significant changes in bone mass measured by both pQCT and histomorphometric analysis in the different appendicular skeleton sites. In line with our results, no detectable changes in the trabecular and cortical bone parameters, as assessed by micro-CT, were shown to be at the bone sites far from the inflamed joints after Ang II administration for four weeks in male mice with Tumor Necrosis Factor-mediated arthritis [45]. Our findings differ from results previously described in experimental models that used chronic administration of Ang II at the same dosage and for the same time used in our experimental model, with subsequent enhancement of bone loss [20,23]. These differences could depend on the different pathophysiologic conditions of animal model applied. In the present study we administered Ang II to male adult Sprague–Dawley rats in physiological conditions. Conversely, in the osteoporosis animal model, Ang II was administered to female animals that had developed ovariectomy induced bone loss [20,23]. It is therefore likely that the gender and the pre-activation of bone remodeling with a negative bone balance might be both critical determinants of RAS activation in bone. Taking into account that in our experimental model we administered Ang II to healthy male Sprague–Dawley rats for four weeks, we can reasonably hypothesize that the duration of the treatment, which was shown to be sufficient to cause changes to other organs, such as the heart [15,17] and kidney [16], is probably too short to cause an alteration in the bone tissue. In fact, in the absence of a pre-activation of bone remodeling characterized by bone loss, it is likely that, to observe a significant modification of bone density, it should be necessary to prolong the duration of administration of Ang II, and possibly also to increase the dosage. Further experimental studies will be needed to verify these hypotheses.

In conclusion, in our experimental model of Ang II dependent hypertension we found changes in urinary calcium/phosphate excretion, which might be due to direct or endocrine mediated mechanisms. In our experimental model, chronic administration of Ang II and/or increased blood pressure values did not affect bone tissue in the absence of pre-activation of bone loss. However, we cannot exclude that, by further extending the experimental time, a detrimental effect of Ang II on bone could become apparent.

## Figures and Tables

**Table 1:** Systolic blood pressure (SBP, mmHg), body weight (BW, g.), glomerular filtration rate (GFR, ml/min), heart/body weight ratio (mg/g), kidney/body weight ratio (mg/g), non-fasting plasma glucose (mg/dL), alkaline phosphatase (U/L), sodium (mEq/L), potassium (mEq/L), calcium (mg/dL), phosphate (mg/dL), uric acid (mg/dL), total cholesterol (mg/dL), triglycerides (mg/dL) in control, control+los, Ang II, Ang II+los-treated rats at the end of the experimental period (four weeks). Data are means ± SEM. * = *p* < 0.05 vs. control; † = *p* < 0.01 vs. control; ‡ = *p* < 0.0001 vs. control; ° = *p* < 0.05 vs. control+los; δ = *p* < 0.01 vs. control+los; § = *p* < 0.0001 vs. control+los; ^ = *p* < 0.05 vs. Ang II+los; & = *p* < 0.01 vs. Ang II+los; ζ = *p* < 0.0001 vs. Ang II+los.

Parameters	Control	Control + Los	Ang II	Ang II + Los
SBP, mmHg	140.5 ± 3.3	135.0 ± 5.0	202.1 ± 3.0 ‡§ζ	142.5 ± 3.2
BW, g	459.1 ± 13.0	433.0 ± 5.2	409.7 ± 13.6 †	423.8 ± 9.4
GFR, ml/min	4.28 ± 0.23	3.58 ± 0.07	3.35 ± 0.36 *	4.15 ± 0.28
Heart/Body Weight, mg/g	2.81 ± 0.06	2.66 ± 0.13	3.93 ± 0.11 ‡§ζ	2.71 ± 0.05
Kidney/Body Weight, mg/g	3.34 ± 0.05	3.51 ± 0.06	4.06 ± 0.09 ‡δζ	3.42 ± 0.13
Plasma				
Glucose (mg/dL)	139± 4.08	142.1 ± 8.15	150.5 ± 5.03	137.2 ± 7.84
Alkaline phosphatase (U/L)	194.3 ± 13.7	194.4 ± 19.5	207.3 ± 6.97	226.7 ± 21.2
Sodium (mEq/L)	140.7 ± 0.38	141.3 ± 0.56	138.0 ± 0.93 †δ^	141.1 ± 1.03
Potassium (mEq/L)	3.95 ± 0.13	4.59 ± 0.33	3.16 ± 0.13 *δ&	4.75 ± 0.54 *
Calcium (mg/dL)	9.83 ± 0.10	9.96 ± 0.09	9.79 ± 0.17	10.0 ± 0.13
Phosphate (mg/dL)	5.72 ± 0.25	6.27 ± 0.46	4.84 ± 0.38 *°^	6.14 ± 0.45
Uric acid (mg/dL)	0.72 ± 0.15	0.72 ± 0.14	0.95 ± 0.18	0.94 ± 0.20
Cholesterol (mg/dL)	71.88 ± 3.96	70.2 ± 4.23	69.31 ± 3.51	71.14 ± 2.09
Triglycerides (mg/dL)	89.81 ± 4.89	98.6 ± 6.86	87.58 ± 21.32	116.34 ± 14.54

**Table 2:** Bone parameters measured by pQCT.

Parameters	Control	Control + Los	Ang II	Ang II + Los
Tb.BMD (mg/cm^3^)	257.0 ± 11.3	277.6 ± 19.7	251.9 ± 9.9	291.7 ± 10.2
Tb.Area (mm^2^)	20.47 ± 1.08	20.80 ± 1.53	21.66 ± 1.07	20.35 ± 0.71
Ct.BMD (mg/cm^3^)	1334.6 ± 4.8	1336.2 ± 8.5	1332.2 ± 6.6	1331.8 ± 5.3
Ct.Area (mm^2^)	4.75 ± 0.16	4.67 ± 0.10	4.52 ± 0.18	4.71 ± 0.16
Tot.Area (mm^2^)	6.91 ± 0.24	6.75 ± 0.19	6.54 ± 0.25	6.90 ± 0.15

Tb.BMD: trabecular bone mineral density, Tb.Area: trabecular area, Ct.BMD: cortical bone mineral density, Ct.Area: cortical area, Tot.Area: total area. Tb.BMD and Tb.Area were measured at the proximal metaphysis of the tibia, Ct.BMD, Ct.Area and Tot.Area were measured at the diaphyseal midshaft. Data are means ± SEM.

## Data Availability

The data presented in this study are available on request from the corresponding author.
